# Prognostic validation and treatment decision making of the 8^th^ edition of the American Joint Committee on Cancer pathological staging system for elderly women with early-stage breast cancer

**DOI:** 10.18632/aging.103574

**Published:** 2020-07-25

**Authors:** San-Gang Wu, Jian Shi, Wen-Wen Zhang, Jun Wang, Chen-Lu Lian, Jian Lei, Li Hua, Juan Zhou, Zhen-Yu He

**Affiliations:** 1 Department of Radiation Oncology, The First Affiliated Hospital of Xiamen University, Teaching Hospital of Fujian Medical University, Xiamen 361003, People’s Republic of China; 2 Department of Breast Surgery, The University of Hong Kong-Shenzhen Hospital, Shenzhen 518083, People’s Republic of China; 3 Department of Radiation Oncology, Sun Yat-Sen University Cancer Center, State Key Laboratory of Oncology in South China, Collaborative Innovation Center of Cancer Medicine, Guangzhou 510060, People’s Republic of China; 4 Department of Obstetrics and Gynecology, The First Affiliated Hospital of Xiamen University, Teaching Hospital of Fujian Medical University, Xiamen 361003, People’s Republic of China

**Keywords:** breast cancer, competing risks model, American Joint Committee on Cancer (AJCC), neoplasm staging

## Abstract

Purpose: To determine the prognostication and treatment decision making of the American Joint Committee on Cancer (AJCC) 8^th^ pathological staging system in elderly women (aged ≥65 years) with T1-2N0M0 breast cancer (BC).

Results: We included 67699 patients, and patients were restaged into stage IA (84.9%), IB (8.9%), and IIA (6.2%) using the 8^th^ AJCC edition criteria. Overall, 69.4% and 30.6% of them underwent breast-conservation surgery (BCS) and mastectomy (MAST), respectively. In patients who received BCS, 30.3% of them underwent postoperative radiotherapy (RT). Patients with a higher pathological stage were more likely to receive MAST. The 5-year breast cancer-specific mortality rate was 2.2%, 6.5% and 13.7% in stage IA, IB, and IIA, respectively. Patients treated with BCS and RT had significantly lower risk of breast cancer-specific mortality compared to those treated with MAST or with BCS alone regardless of the pathological prognostic stages (P<0.001).

Conclusions: The 8^th^ AJCC pathological prognostic staging system provides accurate risk stratification and impacts the treatment decision making for elderly women with early-stage BC.

Methods: We identified stage T1-2N0M0 BC patients using the Surveillance, Epidemiology, and End Results database. Statistical analyses were used binomial logistic regression, and multivariable competing risk models in the Cox model framework.

## INTRODUCTION

Breast cancer (BC) is an age-related disease that mainly affects elderly women (aged ≥65 years), and accounting for approximately 50% of women with newly diagnosed BC [1, 2]. The increasing cancer incidence among elderly women has influenced healthcare planning and delivery [3, 4]. Standard treatment modalities for elderly patients with BC, including surgery, chemotherapy, endocrine therapy, target therapy, and radiotherapy (RT) [5]. Prospective randomized controlled trials have reported similar survival outcomes in patients treated with breast-conservation surgery (BCS) and mastectomy (MAST) [6, 7]. However, elderly patients were largely excluded from these trials. A recent population-based study showed that a larger proportion of BCS-eligible women were treated with MAST [2]. An increased risk of comorbidities, less aggressive biological behavior, and shorter life expectancy might influence the treatment decision making for this population. Therefore, an evidence-based guide to treatment of elderly BC patients is scarce.

The anatomic American Joint Committee on Cancer (AJCC) TNM system (T, tumor; N, nodes; M, metastasis) has been widely used to assess survival outcomes and guide treatment decisions for BC worldwide [8]. Several biologic factors, including tumor grade, estrogen receptor (ER), progesterone receptor (PR), and human epidermal growth factor receptor 2 (HER2) have been integrated into the 8^th^ AJCC pathological prognostic staging system, which provides a better predictive performance for prognosis compared to the 7^th^ AJCC anatomic TNM stages [9–14]. The 7^th^ AJCC anatomic TNM stages does not predict the survival outcome accurately while incorporating biologic factors may refine the risk stratification and have more important role on decision-making of locoregional or systemic treatments. However, whether the new AJCC staging system would provide critical implications to clinicians in making well-informed decisions throughout the course of treatment not yet being validated. Therefore, the role of the pathological prognostic staging system in treatment decision-making for elderly patients with BC should be investigated.

Elderly BC patients have a higher probability of comorbidities, which increase their risk for competing non-cancer events [15]. Failure to explain competing risks in the elderly might lead to misleading conclusions in epidemiological studies or clinical trials [16]. The Kaplan-Meier method or Cox’s proportional-hazards model might not be appropriate for prognostic analyses because they consider competing events as independent censorings and overestimate the proportion of deaths due to cancer. However, the validated studies of the new AJCC pathological prognostic staging system were based on Kaplan-Meier analysis, which considered competing events as independent censorings [11–14]. The competing events are known to be very frequent in the elderly. It is crucial to analyze competitive risks when assessing the prognosis of elderly patients. Therefore, competitive risks analysis may be a more appropriate method because it takes into account the nature of the censoring and corresponds to the probability of a particular event occurring, without having to assume independence between event types [17, 18]. Research on the benefits of treatments for elderly BC patients will help identify effective treatment modalities and ultimately guide optimal treatment decisions. In light of this, we conducted a competing risks analysis to validate the prognostic effect and assess the impact of treatment decision making of the 8^th^ AJCC pathological prognostic stages for elderly BC patients from a large contemporary population-based dataset.

## RESULTS

### Patient characteristics

We identified 67699 patients in this study. Overall, patients with stage IA (78.6%) and IIA (21.4%) diseases in the 7^th^ AJCC edition criteria were restaged in to stage IA (84.9%), IB (8.9%), and IIA (6.2%) diseases using the 8^th^ AJCC pathological staging criteria. The patients’ characteristics are presented in Table 1. Overall, 89.2% of patients resided in the metropolitan area. Most patients in this series were ER-positive (88.4%) and HER2 negative (91.2%). In addition, 78.6% were Non-Hispanic White (NHW) (78.6%), 72.6% were infiltrating ductal carcinoma, 78.6% were T1 stage, 79.2% were well-moderately differentiated, and 77.5% were PR-positive (77.5%). Moreover, 87.3% of them were not received chemotherapy.

**Table 1:** Patients’ baseline characteristics.

**Variables**	**n**	**BCS alone (%)**	**BCS+RT (%)**	**MAST (%)**	**P^a^**	**P^b^**
Age (years)						
65-69	21683	2363 (16.6)	12840 (39.2)	6480 (31.3)	<0.001	0.054
70-74	17435	2942 (20.6)	9139 (27.9)	5354 (25.9)		
75-79	13079	2837 (19.9)	6167 (18.8)	4075 (19.7)		
≥80	15502	6113 (42.9)	4607 (14.1)	4782 (23.1)		
Race/ethnicity						
Non-Hispanic White	53221	11347 (79.6)	26281 (80.2)	15593 (75.4)	<0.001	<0.001
Non-Hispanic Black	5038	1027 (7.2)	2288 (7.0)	1723 (8.3)		
Hispanic (All Races)	4742	1085 (7.6)	2136 (6.5)	1524 (7.4)		
Other	4698	799 (5.6)	2048 (6.3)	1851 (8.9)		
Histological subtype						
IDC	49182	10339 (72.5)	24444 (74.6)	14399 (69.6)	<0.001	<0.001
ILC	6590	1120 (7.9)	2999 (9.2)	2471 (11.9)		
Other	11927	2796 (19.6)	5310 (16.2)	3821 (18.5)		
T stage						
T1	53210	11596 (81.3)	27353 (83.5)	14261 (68.9)	<0.001	<0.001
T2	14489	2659 (18.7)	5400 (16.5)	6430 (31.1)		
Grade						
G1	21812	5337 (37.4)	10975 (33.5)	5500 (26.6)	<0.001	<0.001
G2	31816	6383 (44.8)	15432 (47.1)	10001 (48.3)		
G3	14071	2535 (17.8)	6346 (19.4)	5190 (25.1)		
ER status						
Negative	7866	1305 (9.2)	3476 (10.6)	3085 (14.9)	<0.001	<0.001
Positive	59833	12950 (90.8)	29277 (89.4)	17606 (85.1)		
PR status						
Negative	15224	2825 (19.8)	6827 (20.8)	5572 (26.9)	<0.001	<0.001
Positive	52475	11430 (80.2)	25926 (79.2)	15119 (73.1)		
HER2 status						
Negative	61720	13127 (92.1)	30259 (92.4)	18334 (88.6)	<0.001	<0.001
Positive	5979	1128 (7.9)	2494 (7.6)	2357 (11.4)		
Pathological stages						
IA	57483	12464 (87.4)	28577 (87.3)	16442 (79.5)	<0.001	<0.001
IB	6022	1030 (7.2)	2776 (8.5)	2216 (10.7)		
IIA	4194	761 (5.3)	1400 (4.3)	2033 (9.8)		
Marital status						
Married	33874	5949 (41.7)	17989 (54.9)	9936 (48.0)	<0.001	<0.001
Divorced	7771	1505 (10.6)	3870 (11.8)	2396 (11.6)		
Single	6457	1370 (9.6)	3004 (9.2)	2083 (10.1)		
Widowed	19597	5431 (38.1)	7890 (24.1)	6276 (30.3)		
Chemotherapy						
No/unknown	59115	13206 (92.6)	28303 (86.4)	17606 (85.1)	<0.001	<0.001
Yes	8584	1049 (7.4)	4450 (13.6)	3085 (14.9)		

Abbreviations: BCS, breast-conservation surgery; ER, estrogen receptor; G1, well differentiated; G2, moderately differentiated; G3, poorly/undifferentiated; HER2, human epidermal growth factor receptor 2; IDC, infiltrating ductal carcinoma; ILC, infiltrating lobular carcinoma; PR, progesterone receptor; MAST, mastectomy; RT, radiotherapy; T, tumor.

^a^ BCS alone vs. BCS+RT vs. MAST; ^b^ BCS vs. MAST.

**Figure 1:**
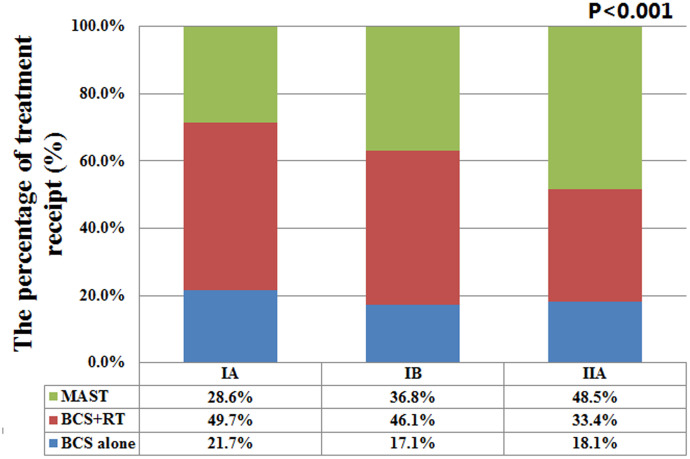
**The percentages of three different local treatments receipt by pathological prognostic stages.**

In the entire cohort, 47008 (69.4%) and 20691 (30.6%) patients underwent BCS and MAST, respectively. In patients who received BCS, 30.3% (n=14255) of them treated with postoperative RT. Significant differences were found in age at diagnosis, race/ethnicity, histology, tumor size, tumor grade, ER status, PR status, HER2 status, pathological prognostic stages, marital status, and chemotherapy (Table 1). In comparison to the elderly women who underwent BCS with or without RT, the patients that underwent MAST included a higher proportion of other races (8.9% vs. 6.3-5.6%), infiltrating lobular cancers (11.9% vs. 7.9%-9.2%), T2 stage (31.1% vs. 16.5-18.7%), poorly/undifferentiated disease (25.1% vs. 17.8-19.4%), ER-negative (14.9% vs. 9.2-10.6%), PR-negative (26.9% vs. 19.8-20.8%), HER2-positive (11.4% vs. 7.6-7.9%), and chemotherapy recipients (14.9% vs. 7.4-13.6%). Patients with a higher pathological prognostic stage (stage IB and IIA) were more likely to be treated with MAST compared to those with stage IA disease. The percentages of treatments received by pathological prognostic stages are presented in Figure 1. Moreover, married patients were more likely to receive BCS and RT, and widowed patients comprised a higher proportion of recipients of BCS alone. No significant difference was found in age between BCS and MAST recipients (P=0.054).

### Stage migration

According to the 7^th^ AJCC staging system, 20.7% (n=13988) of patients were restaged according to the 8^th^ AJCC pathological staging criteria. Overall, 5.5% (n=3693) were upstaged and 15.2% (n=10295) were downstaged (Table 2). Among patients with 7^th^ edition stage IA disease, 6.9% were upstaged to stage IB using the 8^th^ edition criteria. Similarly, 55.0% and 16.1% of the 7^th^ edition stage IIA patients were downstaged to stage IA and IB diseases, respectively.

**Table 2:** Comparison of the 7^th^ and 8^th^ editions of the AJCC breast cancer staging systems.

	**8^th^ ed. stage IA**	**8^th^ ed. stage IB**	**8^th^ ed. stage IIA**	**Total**
7^th^ ed. stage IA	49517 (93.1%)	3693 (6.9%)	0	53210
7^th^ ed. stage IIA	7966 (55.0%)	2329 (16.1%)	4194 (28.9%)	14489
Total	57483 (84.9%)	6022 (8.9%)	4194 (6.2%)	67699

Abbreviation: AJCC, American Joint Committee on Cancer.

Yellow, blue, and grey boxes represent patients whose 7^th^ edition stages were upstaged, down staged, or unchanged, respectively.

### Predictors of the treatments received

We assessed the independent predictors of treatments received using binomial regression with the following variables: histology, tumor stage, pathological stage, race/ethnicity, marital status, and chemotherapy received (Table 3). Non-infiltrating ductal carcinoma, T2 stage, higher pathological stage, non-NHW race, and being unmarried were independent predictors of receipt of MAST.

**Table 3:** Predictors of the surgical procedure received (MAST vs. BCS).

**Variables**	**OR**	**95%CI**	**P**
Race/ethnicity			
Non-Hispanic White	1		
Non-Hispanic Black	1.167	1.096-1.243	<0.001
Hispanic (All Races)	1.119	1.049-1.194	0.001
Other	1.579	1.484-1.681	<0.001
Histological subtype			
IDC	1		
ILC	1.400	1.325-1.479	<0.001
Other	1.126	1.077-1.176	<0.001
Pathological stages			
IA	1		
IB	1.234	1.163-1.309	<0.001
IIA	1.329	1.231-1.433	<0.001
Marital status			
Married	1		
Divorced	1.058	1.002-1.117	0.044
Single	1.098	1.036-1.165	0.002
Widowed	1.089	1.047-1.132	<0.001
Chemotherapy			
No/unknown	1		
Yes	1.096	1.040-1.154	0.001

Abbreviations: BCS, breast-conservation surgery; CI, confidence interval; IDC, infiltrating ductal carcinoma; ILC, infiltrating lobular carcinoma; MAST, mastectomy; OR, odds ratio.

### Survival and prognostic analysis

In the entire cohort, 9177 deaths occurred, but only 20.4% (n=1868) were breast-cancer related. The top five mortality rates were heart disease (25.6%), chronic obstructive pulmonary disease and related conditions (7.6%), cerebrovascular diseases (7.0%), lung and bronchus carcinomas (5.5%), and Alzheimer's disease (4.6%).

**Figure 2:**
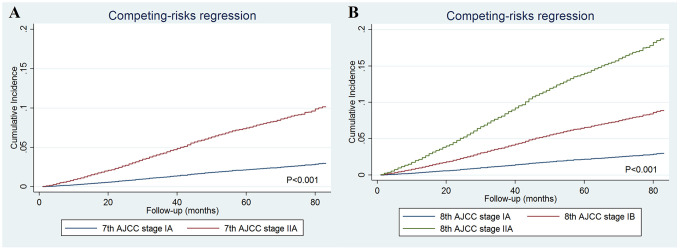
The cumulative incidence estimates of breast cancer-specific mortality rates by the 7^th^ AJCC anatomic staging (**A**) and the 8^th^ AJCC pathological prognostic staging (**B**).

**Figure 3:**
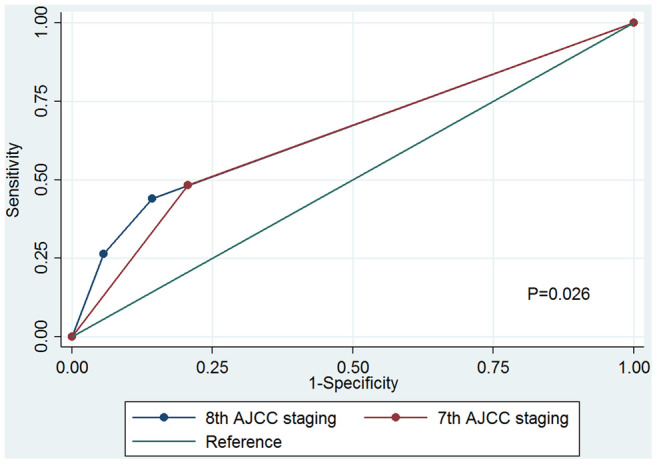
**Receiver operating characteristics analyses for prediction of breast cancer-specific mortality between the 7^th^ AJCC anatomic staging and the 8^th^ AJCC pathological prognostic staging.**

The cumulative incidence estimates of breast cancer-specific mortality (BCSM) by the 7^th^ AJCC staging and the 8^th^ AJCC pathological prognostic staging are presented in Figure 2. Regarding the 7^th^ AJCC staging, the 5-year BCSM rates were 2.2% and 7.4% in patients with stages IA and IIA disease, respectively (P<0.001) (Figure 2A). Patients with a higher pathological stage had a higher cumulative incidence of BCSM. The 5-year BCSM rates were 2.2%, 6.5%, and 13.7% in patients with stages IA, IB, and IIA using the 8^th^ edition criteria, respectively (P<0.001) (Figure 2B). The 8^th^ AJCC staging was examined against the 7^th^ AJCC staging using the receiver operating characteristics (ROC) curve. The area under the curve (AUC) under the ROC curve in 8^th^ AJCC staging (AUC=0.655, 95% confidence interval [CI] 0.643-0.667) was significantly higher than that of the 7^th^ AJCC staging (AUC=0.638, 95%CI 0.627-0.649) (P=0.026) (Figure 3). The results indicated that the 8^th^ AJCC staging had a better predictive performance for BCSM compared to the 7^th^ AJCC staging.

We developed two Gray’s proportional sub-distribution hazards models to investigate the prognostic factors associated with BCSM. The first multivariate prognostic model included biologic factors such as tumor grade, ER, PR, and HER2 status for predicting the BCSM (Table 4). The results showed that higher tumor grade, ER-negative, and PR-negative were the independent adverse prognostic factors related to BCSM. However, HER2 status was not associated with BCSM in the multivariate prognostic analysis. The second multivariate prognostic model included the pathological prognostic staging for predicting BCSM (Table 5). The competing risks model using the Cox model framework showed that pathological prognostic staging was a significant predictor of BCSM. Using stage IA as a reference, the sub-distribution hazard ratios (sdHR) for stages IB and IIA were 2.946 (95%CI 2.598-3.341, P<0.001) and 5.908 (95%CI 5.272-6.620, P<0.001), respectively, compared to stage IA. Age at diagnosis, race/ethnicity, histology, and marital status were also the independent predictors of BCSM (Table 5).

**Table 4:** The multivariate prognostic analysis included biologic factors for predicting the breast cancer-specific mortality using the competing risks model.

**Variables**	**sdHR**	**95% CI**	**P**
Age (years)			
65-69	1		
70-74	1.287	1.117-1.482	<0.001
75-79	1.594	1.379-1.843	<0.001
≥80	2.224	1.946-2.542	<0.001
Race/ethnicity			
Non-Hispanic White	1		
Non-Hispanic Black	1.114	0.955-1.299	0.170
Hispanic (All Races)	1.007	0.837-1.211	0.940
Other	0.740	0.599-0.914	0.005
Histological subtype			
Infiltrating ductal carcinoma	1		
Lobular carcinoma	0.874	0.731-1.046	0.143
Other	0.908	0.797-1.034	0.143
T stage			
T1	1		
T2	2.469	2.242-2.719	<0.001
Grade			
G1	1		
G2	1.613	1.393-1.868	<0.001
G3	2.912	2.478-3.422	<0.001
ER status			
Negative	1		
Positive	0.647	0.559-0.750	<0.001
PR status			
Negative	1		
Positive	0.647	0.567-0.738	<0.001
HER2 status			
Negative	1		
Positive	0.902	0.783-1.038	0.149
Marital status			
Married	1		
Divorce	1.159	0.990-1.356	0.066
Single	1.265	1.076-1.489	0.005
Widowed	1.284	1.150-1.433	<0.001

Abbreviations: CI, confidence interval; ER, estrogen receptor; G1, well differentiated; G2, moderately differentiated; G3, poorly/undifferentiated; HER2, human epidermal growth factor receptor 2; IDC, infiltrating ductal carcinoma; ILC, infiltrating lobular carcinoma; PR, progesterone receptor; sdHR, sub-distribution hazard ratios; T, tumor.

**Table 5:** The multivariate prognostic analysis included 8^th^ AJCC pathological prognostic stages for predicting the breast cancer-specific mortality using the competing risks model.

**Variables**	**sdHR**	**95% CI**	**P**
Age (years)			
65-69	1		
70-74	1.228	1.067-1.414	0.004
75-79	1.467	1.269-1.696	<0.001
≥80	1.921	1.676-2.203	<0.001
Race/ethnicity			
Non-Hispanic White	1		
Non-Hispanic Black	1.171	1.005-1.366	0.044
Hispanic (All Races)	1.006	0.837-1.209	0.951
Other	0.728	0.589-0.900	0.003
Histological subtype			
Infiltrating ductal carcinoma	1		
Lobular carcinoma	0.761	0.639-0.907	0.002
Other	0.830	0.729-0.943	0.004
Pathological stage			
IA	1		
IB	2.946	2.598-3.341	<0.001
IIA	5.908	5.272-6.620	<0.001
Marital status			
Married	1		
Divorce	1.156	0.987-1.352	0.071
Single	1.230	1.045-1.447	0.013
Widowed	1.274	1.142-1.423	<0.001

Abbreviations: CI, confidence interval; sdHR, sub-distribution hazard ratios.

### The effects of the treatment receipt on BCSM

**Figure 4:**
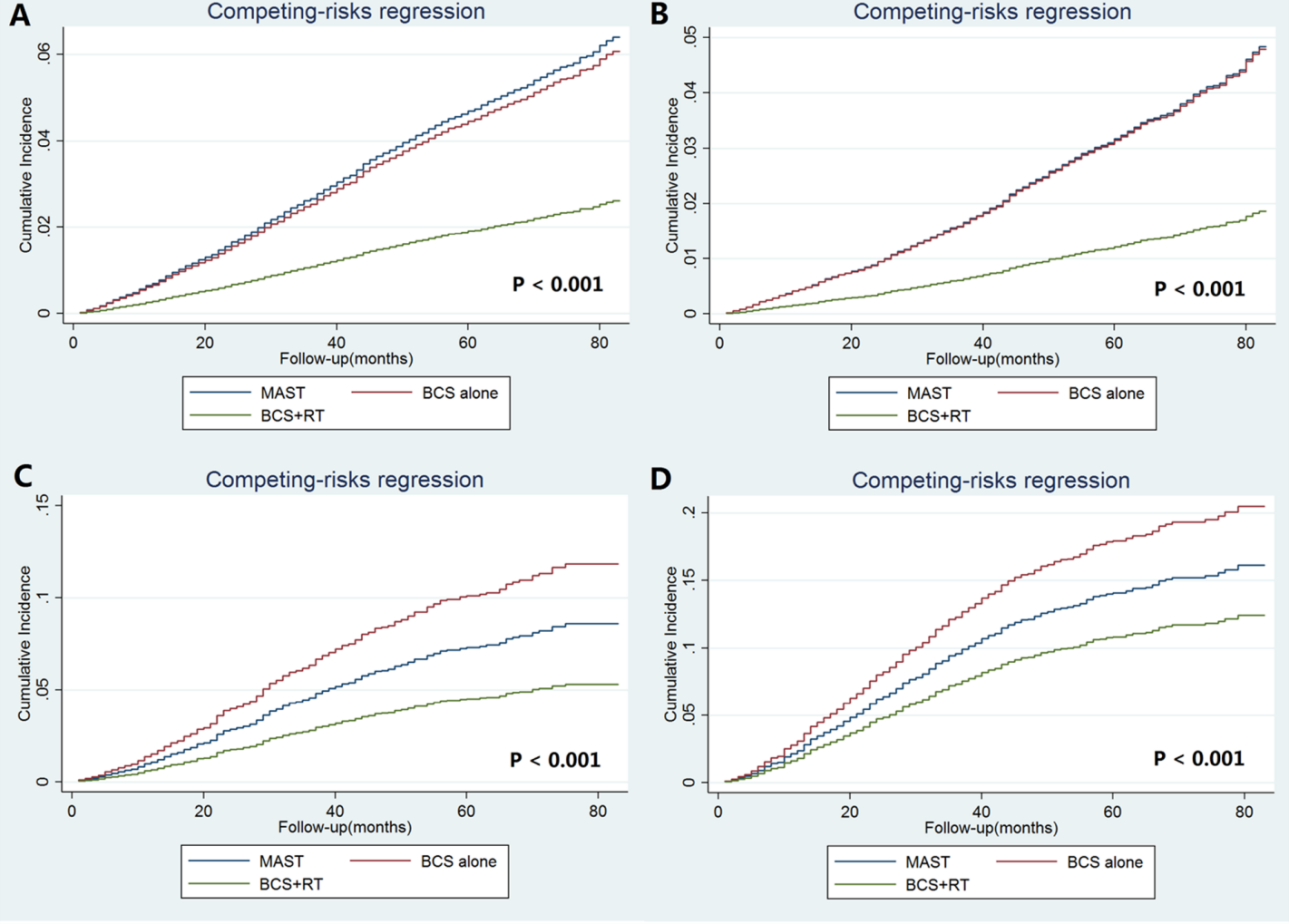
The cumulative incidence estimates of breast cancer-specific mortality rates by local treatment receipt according to the pathological prognostic stages (**A**: entire cohort; **B**: stage IA; **C** stage IB; **D**: stage IIA).

After adjustment of age at diagnosis, race/ethnicity, histology, pathological stages, chemotherapy, and marital status, the results of the competing risks model indicated that local treatment procedure was an independent predictor of BCSM (Table 6). Using MAST as the reference, patients who received BCS alone (sdHR 1.003, P=0.948) had comparable BCSM compared to those treated with MAST, while patients treated with BCS and RT (sdHR 0.520, P<0.001) had significantly lower risk of BCSM than those treated with MAST (Table 6). The cumulative incidence estimates of BCSM by local treatment received are presented in Figure 4A. The rates of the 5-year BCSM were 4.9%, 4.2%, and 1.9% in patients treated with MAST, BCS alone, and BCS with RT, respectively (P<0.001).

**Table 6:** The multivariate prognostic analysis of predictors of breast cancer-specific mortality by local treatment receipt according to pathological prognostic stages.

**Variables**	**sdHR**	**95% CI**	**P**
Entire cohort			
MAST	1		
BCS	1.003	0.894-1.126	0.948
BCS+RT	0.520	0.464-0.583	<0.001
Stage IA			
MAST	1		
BCS	0.858	0.738-0.998	0.047
BCS+RT	0.420	0.362-0.489	<0.001
Stage IB			
MAST	1		
BCS	1.269	0.970-1.660	0.083
BCS+RT	0.632	0.491-0.814	<0.001
Stage IIA			
MAST	1		
BCS	1.212	0.969-1.517	0.092
BCS+RT	0.793	0.638-0.986	0.037

Abbreviations: BCS, breast-conservation surgery; CI, confidence interval; MAST, mastectomy; RT, radiotherapy; sdHR, sub-distribution hazard ratios.

After stratification by pathological prognostic stages, similar survival difference by local treatment received was found in the subgroups of women with stages IB and IIA, adjusting for age at diagnosis, race/ethnicity, histology, chemotherapy, and marital status (Table 6). Regarding the stage IA disease, patients treated with BCS alone (sdHR 0.858, 95%CI 0.738-0.998, P=0.047) and BCS with RT (sdHR 0.420, 95%CI 0.362-0.489, P<0.001) had significantly lower risk of BCSM compared to those treated with MAST (Table 6). The cumulative incidence estimates of BCSM by local treatment receipt after stratification by pathological prognostic stages are presented in Figure 4B–3D.

Finally, the results of the Fine and Gray’s proportional sub-distribution hazard model for BCSM indicated that patients treated with BCS and RT (sdHR 0.521, 95%CI 0.458-0.592, P<0.001) had significantly lower risk of BCSM compared to those treated with BCS alone. Stratified analysis replicated similar findings after stratification according to the pathological prognostic stages (stage IA: sdHR 0.490, 95%CI 0.414-0.579, P<0.001; IB: sdHR 0.497, 95%CI 0.375-0.660, P<0.001; IIA: sdHR 0.654, 95%CI 0.503-0.851, P=0.002).

## DISCUSSION

In this study, we used a population-based database to validate the effect of 8^th^ AJCC pathological staging system on BCSM and treatment decision making in elderly women (aged ≥65 years) with T1-2N0M0 BC. Our results indicated that patients with a higher pathological stage was more likely to have a higher risk of BCSM and more likely to be treated with MAST. Patients treated with BCS and RT had significantly lower risk of BCSM compared to those treated with MAST or with BCS alone regardless of the pathological prognostic stages. The present study has two main merits: the using of the 8^th^ edition of AJCC, and using of a competing risk model instead of Kaplan-Meier and Cox regression analyses of estimating cumulative survival in the elderly.

Given continuous innovations in diagnostic techniques and standard-of-care treatments for BC, recommendations have been made to include tumor grade, ER, PR, and HER2 status in the assessment of the disease’s prognosis and treatment decision-making [19]. The 8^th^ edition of the AJCC Cancer Staging Manual has incorporated these biologic biomarkers into the prognostic staging groups for the first time [9, 10]. Patients aged ≥65 years presented with a higher percentage of ER-positive tumors and a lower percentage of HER2-positive tumors [20, 21]. Therefore, only 20.7% of the patients were restaged in our study, while approximately 50% of patients in all age groups were restaged in other studies [11, 22]. In this study, 71.1% of the patients in stage IIA of the 7^th^ AJCC staging system were downstaged to IA (55.0%) and IB (16.1%) using the 8^th^ edition criteria. We also found patients with a higher pathological stage had a higher cumulative incidence of BCSM using the competing risks model to reduce the potential competitive risk bias in the elderly. Currently, there are no published studies on the assessment of the prognostic effect of the newly pathological prognostic stages for elderly BC patients. Our study indicated that the new pathological prognostic staging system was well suited for the prognostic classification for this population, which could improve the accuracy of predicting outcomes and serve as a guide for optimal adjuvant treatment.

The new edition of the pathological prognostic staging system was developed using data retrieved from the National Cancer Data Base (NCDB) on patients who were mostly treated with appropriate multidisciplinary therapies [9, 10]. A recent study from nine European countries suggested that elderly patients should receive standard medical treatment whenever possible to maximize the benefits of modern evidence-based treatments [23]. Given the increased burden of comorbidities [24], frailty among elderly patients could lead to therapy delays or refusals, which might lead to lower survival rates [25, 26]. In this study, only 20% of deaths were from BC, while cardiovascular, cerebrovascular, and lung diseases were common causes of death in elderly patients. However, life expectancy may be underestimated in elderly BC patients due to better management of their comorbidities [27]. Therefore, although age and comorbidities should be considered in treatment decision making for elderly patients, they should never be considered as obstacles to standard treatment [27, 28].

In the current clinical practice, the 8^th^ pathological prognostic staging alone does not guide treatment decisions that remain based on T stage, N stage, hormone receptor status, HER2 status, and multigene assays, and pathological prognostic staging is valuable in prognostic counseling of patients [29]. However, only limited studies were available regarding the locoregional treatment-decision making of the new staging. A recent study from ours included patients with stage T1-2 and one to three lymph node metastasis (restaged as IA, IB, IIA, IIB, and IIIA according to the 8^th^ AJCC edition criteria), the results showed that postmastectomy radiotherapy was only associated with better BC-specific survival in stage IIIA disease [30]. In addition, we also investigated the treatment decision-making of the new AJCC pathological prognostic staging in T3N0 BC patients (restaged as IA, IB, IIA, IIB, and IIIA according to the 8^th^ AJCC edition criteria), the results showed that postmastectomy radiotherapy was correlated with better BC-specific survival in stage IIB disease [31]. Therefore, the new AJCC staging may also have implications on decisions about locoregional treatment.

Although adjuvant RT may affect decision making about surgical procedures among elderly patients [32], BCS remains the main surgical procedure for this patient group (56-63% with BCS and 37-43% with MAST) [33, 34]. Prospective clinical trials have reported lower local recurrence rates but this finding does not translate into the benefit of distant recurrence-free survival or overall survival rates among elderly women with ER-positive BC [35, 36]. However, the results from the NCDB and a German clinical cohort showed improved survival with additional adjuvant RT following BCS among elderly women [37, 38]. The present study therefore, analyzed the potential role of biologic factors in treatment decision making for this patient group. Our results showed that patients with a higher pathological prognostic stage were more likely to receive MAST, (i.e., 28.6%, 36.8%, and 48.5% of the patients with stage IA, IB, and IIA BC, respectively, were treated with MAST). We also found that MAST was associated with a higher risk of BCSM compared to BCS + RT, and similar findings were replicated after stratification by the pathological prognostic stages. However, among the patients with stages IB and IIA BC, a comparable risk of BCSM was observed in recipients of MAST and BCS alone, indicating that BCS may be an alternative treatment strategy for patients with RT contraindications or those who refuse to receive RT.

Approximately one-third of the patients in this study were not treated with postoperative RT following BCS, which was similar to a previous study [39]. We further analyzed the effect of postoperative RT on BCSM according to pathological prognostic stages. Our results showed that additional postoperative RT was associated with a lower risk of BCSM compared to BCS alone. Concerns about the toxicity of RT might lead practitioners to choose MAST for elderly patients. However, recent studies showed that postoperative RT had no more toxic in elderly women than younger women [28, 40]. A previous population-based cohort study also showed the benefits of postoperative RT for elderly patients did not appear to be influenced by the presence of comorbidities [41]. According to our results, postoperative RT might be considered for elderly patients when their comorbidities are well managed [27, 40, 42].

We used a competing risks model for predicting BCSM in an elderly cohort with a high frequency of competing events. Nevertheless, several inherent limitations should be acknowledged. First, information on selection bias in the choice of treatments by providers and patients was unavailable in the Surveillance, Epidemiology, and End Results (SEER) database. Second, data on comorbidities and functional status were not recorded in the SEER database, which might have an influence on treatment decision making. Moreover, chemotherapy regime, anti-HER2 therapy, and endocrine therapy were also unavailable in the SEER database. Finally, a high rate of under-reporting of RT administration was found in the SEER database.

In conclusion, our study suggests that the 8^th^ AJCC pathological prognostic staging system provides accurate risk stratification and impacts the treatment decision making for elderly women with early-stage BC. Elderly patients with early-stage BC undergoing BCS and RT have lower risk of BCSM than those undergoing MAST or with BCS alone regardless of the pathological prognostic stages. More studies are needed to guide treatment decision making by the new pathological prognostic stages for elderly patients with BC.

## MATERIALS AND METHODS

### Patients

We performed this retrospective analysis, including elderly women (aged ≥65 years) with T1-2N0M0 BC who underwent BCS or MAST between 2010 and 2014. Data on the patients were retrieved from the SEER database. The SEER program is an open-access population-based cancer registry, which including data on tumor incidence, demographic features, tumor characteristics, the first course of treatment, and survival outcomes for approximately 28% of the United States population [43]. We excluded patients with a non-positive pathological diagnosis or insufficient data on race/ethnicity, tumor grade, ER status, PR status, HER2 status, or marital status. This study was exempted from approval by the Institutional Review Board because patients’ information in the SEER database is de-identified.

### Variables

We retrieved the following information for each patient: age, race/ethnicity, tumor stage, tumor grade, histology, ER status, PR status, HER2 status, and marital status. We also collected data on the receipt of surgical procedures, chemotherapy, or postoperative RT for statistical analyses. Patients with stage T1-2N0M0 were reassigned to stages IA, IB, and IIA using the 8^th^ edition of the AJCC pathological prognostic staging manual (9,10).

### Statistical analyses

Patients’ characteristics were compared using the chi-square test, and predictors of receiving local treatments were assessed using binomial logistic regression. Univariate and multivariable competing risks models were used to assess the cumulative incidence of BCSM. The BCSM was defined as the interval from the diagnosis of BC to the date of death from BC. ROC curves were used to assess the discriminatory ability of the 7^th^ AJCC staging system and the 8^th^ AJCC staging system. Competing risks models with the Cox model framework, as proposed by Fine and Gray were used to assess the combined effects of the variables to determine the predictors of BCSM. All statistical analyses were performed using IBM SPSS 22.0 (IBM Corp., Armonk, NY), R statistical software (version 3.5.0; https://www.r-project.org/), and Stata/SE version 14 (StataCorp, TX, USA). All statistical tests were two-tailed, with *P* values <0.05 considered statistically significant.
